# Hydroxyalkyne–Bithiophene Derivatives: Synthesis and Antileishmanial Activity

**DOI:** 10.1111/cbdd.70167

**Published:** 2025-08-22

**Authors:** Rayanne Regina Beltrame Machado, Deysiane Lima Salvador, Carla Maria Beraldi Gomes, Amanda Beatriz Kawano Bakoshi, Tânia Ueda‐Nakamura, Sueli de Oliveira Silva, Celso Vataru Nakamura, Maria Helena Sarragiotto, Danielle Lazarin‐Bidóia

**Affiliations:** ^1^ Laboratório de Inovação Tecnológica no Desenvolvimento de Fármacos e Cosméticos, Departamento de Ciências Básica da Saúde Universidade Estadual de Maringá (UEM) Maringá Brazil; ^2^ Programa de Pós‐Graduação Em Química, Departamento de Química Universidade Estadual de Maringá (UEM) Maringá Brazil

**Keywords:** antileishmanial activity, bithiophenes, cell death, chemical synthesis, mechanism of action

## Abstract

Leishmaniasis is one of the most important neglected tropical diseases, prevalent in underdeveloped or developing countries, and new pharmacological agents for this disease are urgently needed. In this study, thiophene derivatives based on the natural product 5′‐methyl‐(5‐[4‐acetoxy‐1‐butynyl])‐2,2′‐bithiophene were synthesized and evaluated against promastigote forms of *Leishmania amazonensis*. The bithiophene **BT‐1** was the most potent and selective synthetic compound toward the parasites, exhibiting IC_50_ of 23.2 μM against promastigotes and CC_50_ of 216.5 μM against macrophages, and its mechanism of action was determined through biochemical and ultrastructural analyses. An accumulation of lipid bodies, loss of cellular content, increased reactive oxygen species production and lipid peroxidation, damage to the plasma membrane, and mitochondrial depolarization were observed in **BT‐1**‐treated parasites. The results indicated that the death of *L. amazonensis* induced by **BT‐1** occurred via destabilizing the parasite's redox homeostasis. Our results also showed that the synthesis based on the natural compound scaffold consisted of useful strategies to obtain new synthetic antileishmanial compounds.

## Graphical abstract

Novel bithiophene derivatives with activity against *Leishmania amazonensis* were synthesized. All compounds exhibited good bioavailability *in silico*, and among them, **BT‐1** was the most active, inducing redox imbalance in promastigotes.

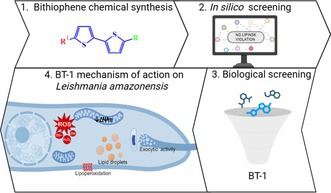

## 1 Introduction

Leishmaniasis is the term used to encompass a group of diseases caused by parasites of the genus *Leishmania* spp., which are transmitted via the bites of infected female phlebotomine sandflies (Pace 2014; De Sarkar et al. 2019). This disease poses a significant public health challenge in many countries, particularly in areas of social and economic fragility. As a result, it falls under the category of neglected tropical diseases (NTDs), conditions for which research funding does not match the global health impact they pose. Annually, an estimated 30,000 to 1 million new cases of leishmaniasis emerge worldwide, with Brazil, East Africa, and India bearing the brunt of the burden (World Health Organization 2023).

The treatment of leishmaniasis is complex and is partly dependent on the causative *Leishmania* species. The current chemotherapeutic arsenal consists of drugs that are clinically approved but not originally developed as antileishmanial agents—for example, miltefosine (initially an anticancer drug), paromomycin (an antibiotic), and amphotericin B (an antifungal). Except for pentavalent antimonials (e.g., Pentostam and Glucantime), most first‐line treatments rely on drug repurposing (Sundar et al. 2025). However, these therapies present significant limitations, including severe toxicity and adverse side effects, the emergence of drug resistance, the need for hospitalization, high treatment costs, and the risk of relapse. As a result, current treatment options for leishmaniasis remain unsatisfactory (Singh et al. 2016; Gupta et al. 2023). Therefore, in this scenario, it is essential to find new alternative compounds that are less toxic and more effective for treating patients with leishmaniasis.

In this context, studies with several classes of compounds for the development of new antileishmanial agents were described (Orosco et al. 2024; Pal et al. 2024). Natural products from plants have been investigated under different approaches, including a focus on employing natural antileishmanial scaffolds in the discovery of new synthetic drugs for leishmaniasis (Pal et al. 2024). In previous work of our research group, thiophene derivatives with antileishmanial activity were isolated from *Porophyllum ruderale* (Jacq.) Cass. The compound 5′‐methyl–(5–[4–acetoxy‐1–butynyl])–2,2′‐bithiophene exhibited significant antileishmanial activity against parasites of *Leishmania amazonensis* (Takahashi et al. 2011).

The thiophene nucleus is an important scaffold present in natural products and synthetic derivatives and has attracted attention in the medicinal field due to its diverse biological activities including antileishmanial, antiviral, antimicrobial, anti‐inflammatory, larvicidal, antioxidant, insecticidal, cytotoxic, and nematicidal (de Lima Serafim et al. 2018; Rodriguez et al. 2018; Singh et al. 2020; Ibrahim et al. 2022; Bigot et al. 2023; Mishra et al. 2025). The potential of bithiophene derivatives has also been demonstrated for our research group (Jacomini et al. 2016; Volpato et al. 2018; Scariot et al. 2019; Rosa et al. 2021). Besides this, Schiff bases were reported to possess antileishmanial activities (Pawar et al. 2023). Also, thiosemicarbazone derivatives have been described as potent antileishmanial compounds (da Silva et al. 2024).

**FIGURE 1:**
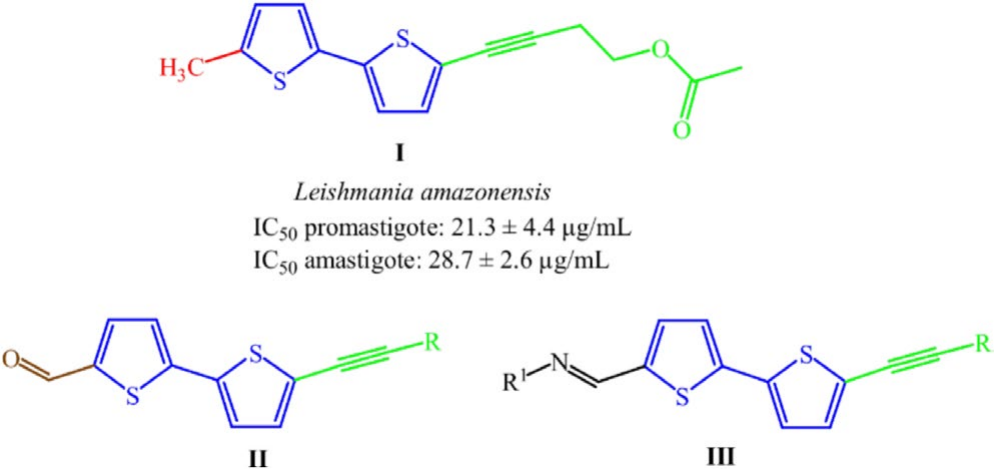
Chemical structures and antileishmanial activity data of natural product (I) obtained in previous work, and of those proposed in this work (II and III).

Taking into account the mentioned, the antileishmanial potential of thiophenes, and based on the structure of the natural product 5′‐methyl‐(5‐[4‐acetoxy‐1‐butynyl])‐2,2′‐bithiophene (**I**) (Figure 1) isolated in our previous work (Takahashi et al. 2011), the 5′‐(hydroxy‐alkynyl)‐bithiophenes (**II**) and a series of bithiophene‐based derivatives (**III**) were synthesized and evaluated against promastigote forms of *L. amazonensis*. The mechanism of action of the most active compound was also evaluated.

## 2 Materials and Methods

### 2.1 Chemicals

#### 2.1.1 Chemistry

All reagents for synthesis were purchased from Sigma‐Aldrich (St. Louis, MO, USA). The reactions were monitored by thin layer chromatography (TLC) using silica gel 60 F_254_ TLC plates (Merck, Darmstadt, Germany). ^1^H NMR and ^13^C‐NMR spectra were recorded in a Varian spectrometer model Mercury plus BB at 300 MHz and 75 MHz, and in a Bruker spectrometer model Avance III HD at 500 MHz and 125 MHz, using DMSO‐*d*_*6*_ and CDCl_3_ as solvent. Mass spectra (ESI/MS) were recorded on Thermoelectron Corporation Focus‐DSQ II spectrometer. Melting points were determined in Microquímica apparatus model MQAPF‐301 and are uncorrected.

#### 2.1.2 Biological Analysis

Actinomycin D, 5ʹ‐bromo‐(2,2ʹ‐bithiophene)‐5‐carboxaldehyde (C_9_H_5_BrOS_2_), butan‐1‐amine, carbonyl cyanide m‐chlorophenylhydrazone (CCCP), calcium chloride (CaCl_2_), cyclohexanamine, copper (I) iodide (CuI), digitonin, dimethyl sulfoxide (DMSO), diphenyl‐1‐pyrenylphosphine (DPPP), 2ʹ,7ʹ‐dichlorodihydrofluorescein diacetate (H_2_DCFDA), Nile red, penicillin, rhodamine 123 (Rh123), 2,3‐bis‐(2‐methoxy‐4‐nitro‐5‐sulfophenyl)‐2H‐tetrazolium‐5‐carboxanilide (XTT), and 3‐(4,5‐dimethylthiazol‐2‐yl)‐2,5‐diphenyltetrazolium bromide formazan (MTT) were purchased from Sigma‐Aldrich (St. Louis, MO, USA). Fetal bovine serum (FBS) was obtained from Invitrogen (Grand Island, NY, USA). Propidium iodide (PI) and RNase were obtained from Invitrogen (Eugene, OR, USA). Cacodylate buffer, glutaraldehyde, potassium ferricyanide (K_4_[Fe(CN)_6_]), osmium tetroxide (OsO_4_), and Poly/Bed 812 resin were obtained from Electron Microscopy Sciences (Hatfield, PA, USA). All reagents were of analytical grade.

### 2.2 Synthesis of 5′‐(Hydroxy‐Alkynyl)‐[2,2′‐Bithiophene]‐5‐Carbaldehydes (1–3)

**SCHEME 1:**
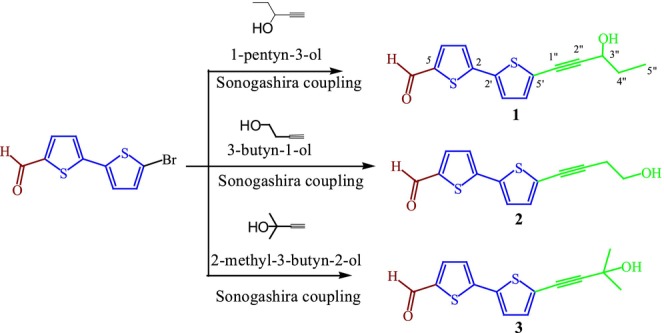
Synthesis of **1–3**. Sonogashira coupling conditions: 6.5 eq. of alkyne, 10 mol% of Pd(PPh_3_)_2_Cl_2_, 20 mol% of CuI, 6.5 eq. of triethylamine, DMF, 70°C, 6 h.

To a solution of commercial 5ʹ‐bromo‐(2,2ʹ‐bithiophene)‐5‐carboxaldehyde (0.37 mmol), Pd(PPh_2_)_3_Cl_2_ (10 mol%), CuI (20 mol%), triethylamine (330 μL, 2.38 mmol) in dimethylformamide (2 mL) was added pent‐1‐yn‐3‐ol, 3‐butyn‐1‐ol, or 2‐methyl‐3‐butyn‐2‐ol (2.38 mmol, 6.5 equivalents) at 25°C under N_2_ atmosphere. The reaction mixture was stirred at 70°C for 6 h. After cooling, distilled water (10 mL) was added, and the solution was extracted with ethyl acetate (3 × 15 mL). The organic layer was dried with anhydrous sodium sulfate, filtered, and evaporated under vacuum. The crude product was subjected to a silica gel 60 chromatographic column using hexane/ethyl acetate 20% to 80% to afford the final products (**1–3**) (Scheme 1).

### 2.3 Synthesis of Bithiophene‐Imines (2a–c) and (3a–c)

**SCHEME 2:**
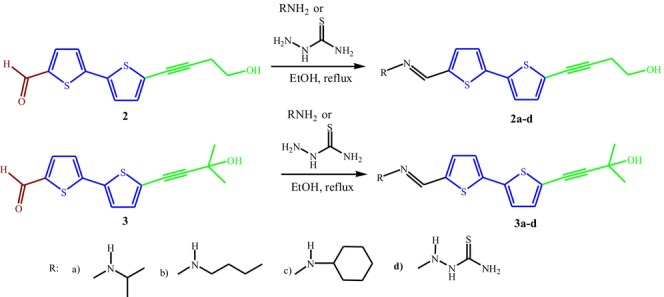
Synthesis of bithiophene‐imines **2a–c** and **3a–c**, and bithiophene‐thiosemicarbazones **2d** and **3d**.

To a solution of 5′‐(4‐hydroxy‐but‐1‐ynyl)‐[2,2′]bithiophenyl‐5‐carbaldehyde (**2**) or 5′‐(3‐hydroxy‐3‐methyl‐but‐1‐ynyl)‐[2,2′]bithiophenyl‐5‐carbaldehyde (**3**) (0.11 mmol) in ethanol (10 mL) was added the appropriate amine (6 eq). The mixture was refluxed under stirring, and the progress of the reaction was accompanied by TLC. After consumption of the reactants (48–72 h), the reaction mixture was cooled and the precipitate formed was filtered, washed with ice water, and dried, providing the corresponding derivatives **2a–c** and **3a–c**, respectively. To obtain the compound **3a**, 10 mol% of Zinc‐proline (Zn(L‐Pro)_2_) was added as a catalyst (0.0032 g, 0.011 mmol) (Scheme 2).

### 2.4 Synthesis of Thiosemicarbazones (2d and 3d)

To a solution of 5′‐(4‐hydroxy‐but‐1‐ynyl)‐[2,2′]bithiophenyl‐5‐carbaldehyde (**2**) or 5′‐(3‐hydroxy‐3‐methyl‐but‐1‐ynyl)‐[2,2′]bithiophenyl‐5‐carbaldehyde (**3**) (0.0300 g; 0.11 mmol) in ethanol (10 mL) was added thiosemicarbazide (0.0200 g; 0.22 mmol). The mixture was refluxed under stirring for 5 h. After cooling, the formed precipitate was filtered, washed with ice water, and dried (Scheme 2).

### 2.5 *In Silico* Physicochemical Characterization

Absorption, distribution, metabolism, excretion (ADME), permeability, and flexibility parameters were evaluated using the free online software SwissADME. The molecular structures were drawn, and a Simplified Molecular Input Entry System (SMILES) code for each molecule was generated. Key parameters were obtained, including molecular weight (MW), hydrophobicity (Consensus logP, average of different predictive methods: iLOGP, XLOGP3, WLOGP, MLOGP, and SILICOS‐IT), the number of hydrogen bond donors and acceptors, topological polar surface area, and rotatable bonds. Lipinski's Rule of Five was used as a parameter for comparison.

### 2.6 Parasite and Macrophage Culture

*L. amazonensis* MHOM/BR/75/Josefa strain was originally isolated from a human case of CL by Dr. Cesar *A. Cuba* at the University of Brasilia, Brazil (Saraiva et al. 1983; Cuba et al. 1985). Promastigote forms of this strain were maintained axenically in Warren's medium (brain–heart infusion plus hemin and folic acid; pH 7.2) supplemented with 10% heat‐inactivated FBS and maintained at 25°C. J774A.1 macrophages (obtained from the Cell Bank of Rio de Janeiro, RJ, Brazil) were maintained in RPMI‐1640 medium (pH 7.2), supplemented with sodium bicarbonate, L‐glutamine, 10% FBS, and streptomycin and penicillin at 37°C in a 5% CO_2_ atmosphere.

### 2.7 Biological Activity of Compounds

#### 2.7.1 Antiproliferative Assay

*L. amazonensis* promastigote forms (1 × 10^6^ parasites.mL^−1^) were cultured in 96‐well plates containing Warren's medium supplemented with 10% FBS in the absence or presence of different concentrations (3.125–100 μM) of the compounds and incubated at 25°C for 72 h. The activity against promastigotes was evaluated by XTT (0.5 mg mL^−1^), as previously described (Meshulam et al. 1995). Absorbance was read in a microplate spectrophotometer (PowerWave XS; BioTek, Winooski, VT, USA) at 450 nm. The concentrations of the compounds that inhibited 50% of parasite growth (IC_50_) were calculated using logarithmic regression analysis.

#### 2.7.2 Cytotoxicity Assay in Macrophages

Cytotoxicity was evaluated in the J774A.1 macrophages. Macrophages (5 × 10^5^ cells mL^−1^) were cultured in 96‐well microplates containing RPMI‐1640 medium supplemented with 10% FBS. The plates were incubated at 37°C in a 5% CO_2_ atmosphere for confluent growth of the cells. After 24 h, the compounds were added to each well at increasing concentrations (31.25–1000 μM) and incubated for 48 h at 37°C in a 5% CO_2_ atmosphere. The cytotoxicity was evaluated by MTT (2 mg mL^−1^), as previously described (Mosmann 1983). Absorbance was read in a microplate spectrophotometer (PowerWave XS; BioTek, Charlotte, VT, USA) at 570 nm. The 50% cytotoxicity concentration (CC_50_) was determined by logarithmic regression analysis of the data obtained.

### 2.8 Mechanism of Action Evaluation

#### 2.8.1 Morphological and Ultrastructural Changes

Promastigote forms (1 × 10^6^ parasites mL^−1^) were treated with the IC_50_ or 2 × IC_50_ of **BT‐1** for 72 h at 25°C. After this, the parasites were fixed in a solution of 2.5% glutaraldehyde in 0.1 M cacodylate buffer for 24 h at 4°C. For scanning electron microscopy (SEM), the parasites were dehydrated in increasing concentrations of ethanol, critical point‐dried in CO_2_, sputter‐coated with gold, and observed in a Quanta 250 scanning electron microscope (FEI Company, Hillsboro, OR, USA). For transmission electron microscopy (TEM), the parasites were fixed as described for SEM. After this, the parasites were postfixed in a solution of 1% OsO_4_, 0.8% K_4_[Fe(CN)_6_], and 10 mM CaCl_2_ in 0.1 M cacodylate buffer. The samples were dehydrated in increasing concentrations of acetone and embedded in Poly/Bed 812 resin. Ultrathin sections were then obtained, contrasted with uranyl acetate and lead citrate, and observed in a JEM 1400 transmission electron microscope (JEOL Ltd., Tokyo, Japan).

#### 2.8.2 Reactive Oxygen Species (ROS) Production

Promastigotes at 1 × 10^6^ parasites mL^−1^ treated with **BT‐1** for 24 h were incubated with 10 μM H_2_DCFDA in the dark for 45 min. As a positive control, 50 μM H_2_O_2_ was used. The fluorescence was determined by oxidation of H_2_DCFDA to the fluorescent product, 2′‐7ʹ dichlorofluorescein (DCF), which was measured in a Victor X3 spectrofluorimeter (PerkinElmer, Waltham, MA, USA) at λ_ex_ of 488 nm and λ_em_ of 530 nm (Shukla et al. 2012).

#### 2.8.3 Lipid Peroxidation Assay

Promastigotes at 1 × 10^6^ parasites mL^−1^ treated with **BT‐1** for 24 h were incubated with 50 μM DPPP for 15 min at 22°C. As a positive control, 50 μM H_2_O_2_ was used. The fluorescence was determined in a Victor X3 spectrofluorimeter (PerkinElmer, Waltham, MA, USA) at λ_ex_ of 355 nm and λ_em_ of 460 nm. DPPP is essentially nonfluorescent until it is oxidized to a phosphine oxide (DPPP‐O) by peroxides (Okimoto et al. 2000).

#### 2.8.4 Mitochondrial Membrane Potential (∆Ψm)

Promastigotes at 1 × 10^6^ parasites mL^−1^ treated with **BT‐1** for 24 h were incubated with 5 μg mL^−1^ Rh123, a fluorescent probe that accumulates in mitochondria, for 15 min (Menna‐Barreto, Corrêa, et al. 2009; Menna‐Barreto, Goncalves, et al. 2009). As a positive control, 100 μM CCCP was used. The data acquisition and analysis were performed using a FACSCalibur flow cytometer (BD Biosciences, Franklin Lakes, NJ, USA) equipped with CellQuest software. A total of 10,000 events were acquired in the region corresponding to the parasites.

#### 2.8.5 Cell Membrane Integrity

Promastigotes at 1 × 10^6^ parasites mL^−1^ treated with **BT‐1** for 24 h were incubated with 0.2 μg mL^−1^ PI for 10 min. As a positive control, 40 μM digitonin was used. The data acquisition and analysis were performed using a FACSCalibur flow cytometer (BD Biosciences, Franklin Lakes, NJ, USA) equipped with CellQuest software. A total of 10,000 events were acquired in the region that corresponded to the parasites. Alterations in PI fluorescence were quantified as the percentage increase in fluorescence compared with the untreated parasites (Lazarin‐Bidóia et al. 2013).

#### 2.8.6 Cell Size

Promastigotes at 1 × 10^6^ parasites mL^−1^ treated with **BT‐1** for 24 h were washed twice and resuspended in phosphate‐buffered saline (PBS), then analyzed using a FACSCalibur flow cytometer (BD Biosciences, Franklin Lakes, NJ, USA) equipped with CellQuest software. As a positive control, 20 mM actinomycin D was used. A total of 10,000 events were acquired in the region that corresponded to the parasites. Histograms were generated for each sample, where the forward light scatter (FSC‐H) represents the cell volume (Lazarin‐Bidóia et al. 2013).

#### 2.8.7 Cell Cycle

Promastigotes at 1 × 10^6^ parasites mL^−1^ treated with **BT‐1** for 24 h were fixed in 70% cold methanol in PBS at 4°C for 2 h. Afterward, the parasites were washed in PBS, 10 μg mL^−1^ PI‐RNAse A was added, and the samples were incubated at 37°C for 45 min. The data acquisition and analysis were performed using a FACSCalibur flow cytometer (BD Biosciences, Franklin Lakes, NJ, USA) equipped with CellQuest software. A total of 10,000 events were acquired in the region that corresponded to the parasites. As a positive control, 40 μM miltefosine was used. The percentages of cells in each stage of the cell cycle were determined based on the intensity of the PI fluorescence, which is directly proportional to the DNA content.

#### 2.8.8 Lipid Bodies

Promastigotes at 1 × 10^6^ parasites mL^−1^ treated with **BT‐1** for 24 h were incubated with 10 μg mL^−1^ Nile red, a lipophilic stain, for 30 min at 25°C. The fluorescence was measured in a Victor X3 spectrofluorimeter (PerkinElmer, Waltham, MA, USA) at λ_ex_ of 485 nm and λ_em_ of 535 nm (Stefanello et al. 2014).

### 2.9 Statistical Analysis

The data are expressed as the means and standard deviations from at least three independent experiments. The data were analyzed using one‐ and two‐way analysis of variance (ANOVA); significant differences among means were identified by Tukey and Bonferroni post hoc tests, respectively. *p* values < 0.05 were considered statistically significant. The statistical analyses were performed using Prism 5 software (GraphPad, San Diego, CA, USA).

## 3 Results

### 3.1 Chemistry

Compounds **1**–**3** were synthesized from the Sonogashira coupling reaction of the commercial 5ʹ‐bromo‐(2,2ʹ‐bithiophene)5‐carboxaldehyde with 1‐pentyn‐3‐ol, 3‐butyn‐1‐ol, or 2‐methyl‐3‐butyn‐2‐ol, using 10 mol% of (Pd(PPh_3_)_2_Cl_2_) as catalyst, 20 mol% of CuI as co‐catalyst, triethylamine as base, and dimethylformamide as solvent at 70°C under nitrogen atmosphere (Scheme 1). The spectral data of compounds synthesized are presented in the Data S1.

Besides the (hydroxy‐alkynyl)‐bithiophene carbaldehydes (**1–3**), a series of bithiophene‐imines **2a–c** and **3a–c**, as well as the bithiophene‐thiosemicarbazones **2d** and 3d were also synthesized from the condensation reaction of intermediates **2** and **3**, respectively, with different amines or thiosemicarbazide in refluxing ethanol (Scheme 2). The spectral data of compounds synthesized are presented in the Data S1.

### 3.2 *In Silico* Characterization

To evaluate the bioavailability of the synthesized bithiophenes and minimize the off‐target binding and promiscuity in vitro and in vivo next assays, we performed the ADME, polar surface area, and rotatable bonds *in silico* analysis. The data for bithiophene derivatives are outlined in Table 1.

**TABLE 1:** *In silico* prediction of bioavailability of bithiophenes compounds.

Compounds	MW (g/mol)	LogP	H acceptors	H donors	Lipinski violation	TPSA (Å^2^)	RB
**1**	276.37	3.44	2	1	NO	93.78	3
**2**	278.39	2.94	2	1	NO	93.78	3
**3**	276.37	3.36	2	1	NO	93.78	2
**2a**	303.44	4.15	2	1	NO	89.07	4
**2b**	317.47	4.53	2	1	NO	89.07	6
**2c**	343.51	4.91	2	1	NO	89.07	4
**2d**	335.47	2.99	2	3	NO	159.21	5
**3a**	317.47	4.33	2	1	NO	89.07	3
**3b**	331.50	4.71	2	1	NO	89.07	5
**3c**	357.53	5.09	2	1	NO	89.07	3
**3d**	349.49	3.19	2	3	NO	159.21	4
**I**	290.40	4.21	2	0	NO	82.78	4

Abbreviations: LogP, logarithm of the partition coefficient; MW, molecular weight; RB, rotatable bonds; TPSA, topological polar surface area.

From the data, it was observed that none of the compounds violated more than 1 rule of Lipinski's RO5, meaning all of them are orally bioavailable. Compounds 2d and 3d present high topological polar surface area (TPSA), which could indicate reduced permeability, potentially hindering drug ability. Despite 3c having a higher logP value, it is still considered bioavailable, as none of the other rules are violated. Following these parameters, most synthetic compounds analyzed in this study had good ADME properties. These findings encouraged us to study the effects caused in vitro by bithiophenes on parasites.

### 3.3 Biological Assays

#### 3.3.1 Antiproliferative Activity Against *L. amazonensis*

The results of assays against *L. amazonensis* for bithiophene derivatives are shown in Table 2. The IC_50_ values ranged from 23.2 to 94.0 μM and CC_50_ values ranged from 198.4 to 295.5 μM. Considering the relationship between the activity against *L. amazonensis* promastigotes and the cytotoxicity against J774A.1 macrophage, the SI ranged from 3.00 to 9.33 (Table 2). The Schiff base derivatives **2a–c** and **3a–c**, as well as the thiosemicarbazones **2d** and **3d** were less active than the (hydroxy‐alkynyl)‐bithiophene carbaldehydes **1** and **2**. The nature of the substituent at the nitrogen atom of **2a–d** and **3a–d** caused no significant variation in IC_50_ values. Amphotericin B, used as a reference compound, showed the lowest IC_50_ and consequently the highest SI.

**TABLE 2:** Evaluation of the antiproliferative activity against *L. amazonensis* and cytotoxicity on J774A.1 macrophages of the synthetic bithiophenes.

Compound	*L. amazonensis* (promastigotes) IC_50_ (μM)	Macrophages J774A.1 CC_50_ (μM)	Selectivity index (SI)
**1**	23.2 ± 1.1	216.5 ± 5.1	9.33
**2**	25.1 ± 0.8	198.4 ± 3.9	7.90
**3**	94.0 ± 4.2	282.9 ± 6.7	3.00
**2a**	44.1 ± 2.0	289.4 ± 6.5	6.56
**2b**	48.5 ± 4.2	274.3 ± 5.2	5.65
**2c**	51.2 ± 1.5	270.7 ± 2.8	5.28
**2d**	41.4 ± 0.6	224.3 ± 4.9	5.42
**3a**	49.5 ± 3.1	281.0 ± 6.2	5.68
**3b**	55.3 ± 5.1	295.5 ± 4.1	5.34
**3c**	40.1 ± 1.1	238.5 ± 1.9	5.95
**3d**	39.5 ± 5.1	219.6 ± 3.8	5.56
Amphotericin B	0.06 ± 0.0	3.7 ± 0.3	61.7

*Note:* SI: selectivity index, the ratio between the CC_50_ and IC_50_.

Among the synthesized compounds, the bithiophene 1 (**BT‐1**) emerged as the most potent, with an IC_50_ of 23.2 μM against *L. amazonensis* promastigotes and no violation of Lipinski's rule of five. Importantly, **BT‐1** showed no toxicity to J774A.1 macrophages at this concentration, presenting a good selectivity index (SI). Even though the **BT‐1** SI is lower than the SI of the natural product (Takahashi et al. 2011), **BT‐1** was shown to be the most potent and selective synthetic molecule toward parasites. The compound was also rapidly obtained in one step from a commercial starting material in a 73% yield. Therefore, further studies were conducted with this compound to determine its mechanism of action.

#### 3.3.2 BT‐1 Induces Redox Imbalance and Ultrastructural Alterations in *L. amazonensis*

**FIGURE 2:**
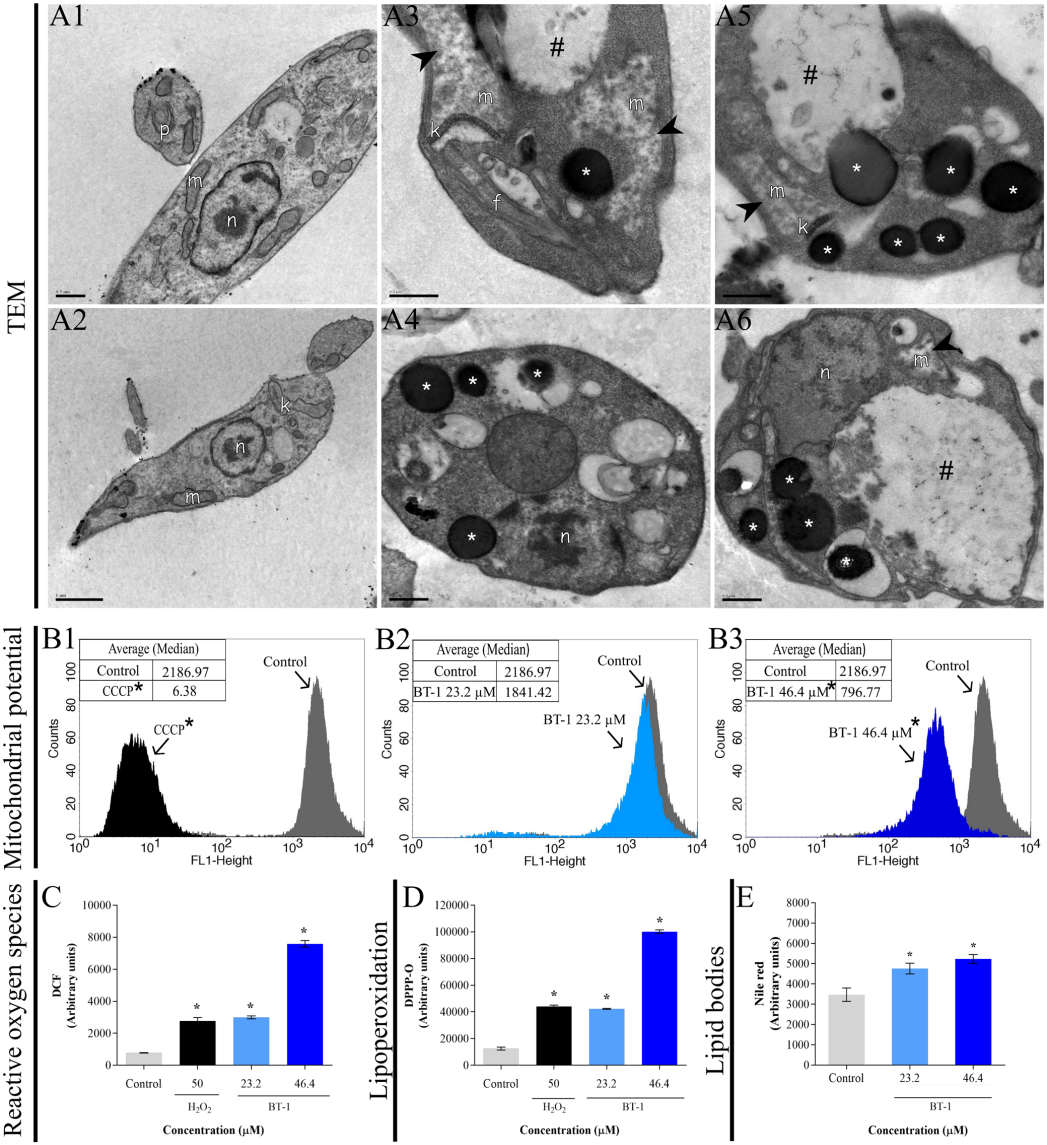
Oxidative stress and ultrastructural alterations in promastigotes of *L. amazonensis* treated with **BT‐1**. (A) Transmission electron microscopy of: Untreated parasites (A1‐2); parasites treated with IC_50_ of **BT‐1** for 72 h (A3‐4); parasites treated with 2 × IC_50_ of **BT‐1** for 72 h (A5‐6). (n) nucleus; (m) mitochondrion; (p) flagellar pocket; (k) kinetoplast; (f) flagellum; (*) lipid vacuoles; (#) vacuoles; (►) mitochondrion swelling. Scale bar: 1 μm (A1); 0.5 μm (A2‐6). (B) Histograms of the mitochondrial membrane potential of: Untreated parasites overlayed with parasites treated with CCCP for 24 h (B1); untreated parasites overlayed with parasites treated with IC_50_ of **BT‐1** for 24 h (B2); untreated parasites overlayed with parasites treated with 2 × IC_50_ of **BT‐1** for 24 h (B3). (C) Reactive oxygen species of parasites treated with IC_50_ and 2 × IC_50_ of **BT‐1** for 24 h. (D) Lipoperoxidation in parasites treated with IC_50_ and 2 × IC_50_ of **BT‐1** for 24 h. (E) Lipid bodies accumulation in parasites treated with IC_50_ and 2 × IC_50_ of **BT‐1** for 24 h. * Indicates a significant difference relative to the control (untreated) group (*p* < 0.05). All the experiments were performed with *n* = 3.

To investigate the mechanism of action of **BT‐1** on promastigotes of *L. amazonensis*, TEM was performed, and based on its results, ROS, lipoperoxidation, lipid bodies accumulation, and mitochondrial potential were analyzed. Analysis of the ultrastructure by TEM revealed the disturbance caused by oxidative stress as the presence of lipid bodies, loss of cell content, disorganization of the nucleus, a large amount of exocytic activity, and mitochondrial swelling in *L. amazonensis* promastigotes (Figure 2A3–A6), while untreated parasites exhibited well‐preserved structures (Figure 2A1–A2). A dose‐dependent decrease in mitochondrial potential was also observed when parasites were treated with **BT‐1** for 24 h. 2.75‐fold of mitochondrial potential has been reduced after the treatment with 2 × IC_50_, compared with the untreated control, indicating mitochondrial depolarization (Figure 2B3). CCCP acted as a mitochondrial uncoupling agent with a reduction of 342.8‐fold in the mitochondrial membrane potential (Figure 2B1).

Additionally, promastigotes treated with the IC_50_ and 2 × IC_50_ concentrations of **BT‐1** resulted in a significant dose‐dependent increase in the production of total ROS, 3.0‐ and 9.0‐fold, respectively (Figure 2C) and in the lipoperoxidation, 3.0‐ and 8.0‐fold, respectively (Figure 2D), when compared to the untreated control group. H_2_O_2_ also caused an increase in the production of ROS and lipoperoxidation of 3.5‐ and 3.6‐fold, respectively. The increase in lipid bodies in **BT‐1**‐treated promastigotes was evaluated further, and the results confirmed the formation and accumulation of lipid bodies after treatment with the IC_50_ and 2 × IC_50_, compared with the untreated control. A 1.4‐ and 1.5‐fold increase in lipid body accumulation was observed after treatment with the IC_50_ and 2 × IC_50_, respectively (Figure 2E).

**FIGURE 3:**
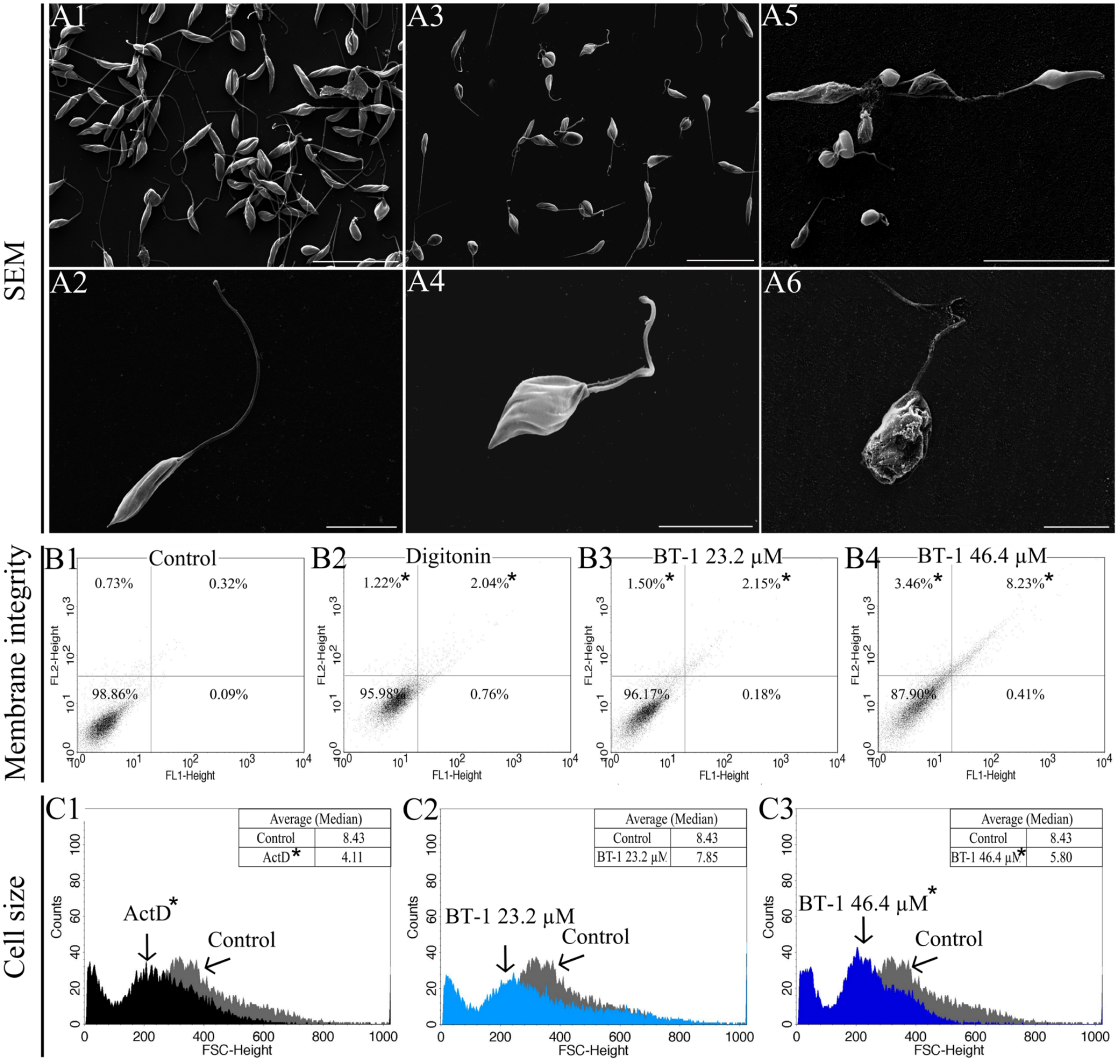
Morphological changes in promastigotes of *L. amazonensis* treated with **BT‐1**. (A) Scanning electron microscopy of: Untreated parasites (A1‐2); parasites treated with IC_50_ of **BT‐1** for 72 h (A3‐4); parasites treated with 2 × IC_50_ of **BT‐1** for 72 h (A5‐6). Scale bar: 20 μm (A1, A3, A5); 5 μm (A2, A4, A6). (B) Membrane integrity of: Untreated parasites (B1); parasites treated with digitonin for 5 min (B2); parasites treated with IC_50_ of **BT‐1** for 24 h (B3); parasites treated with 2 × IC_50_ of **BT‐1** for 24 h (B4). (C) Histograms of the cell size of: Untreated parasites overlayed with parasites treated with actinomycin D for 24 h (C1); untreated parasites overlayed with parasites treated with IC_50_ of **BT‐1** for 24 h (C2); untreated parasites overlayed with parasites treated with 2 × IC_50_ of **BT‐1** for 24 h (C3). *Indicates a significant difference relative to the control (untreated) group (*p* < 0.05). All the experiments were performed with *n* = 3.

**FIGURE 4:**
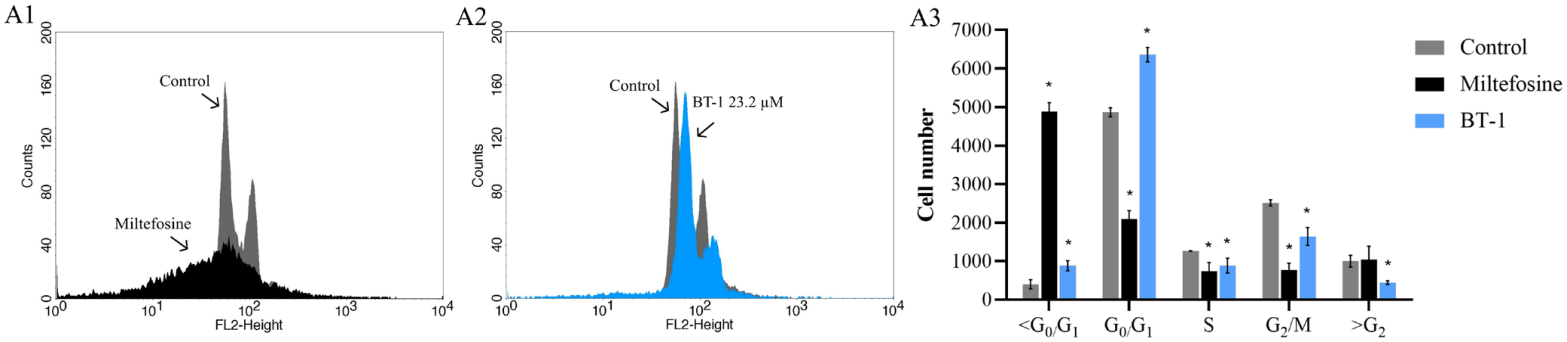
Cell cycle of promastigotes of *L. amazonensis* treated with **BT‐1**. (A) Histograms of untreated parasites overlayed with parasites treated with miltefosine for 24 h (A1); untreated parasites overlayed with parasites treated with IC_50_ of **BT‐1** for 24 h (A2); Statistical analysis of cell cycle (A3). * Indicates a significant difference relative to the control (untreated) group (*p* < 0.05). All the experiments were performed with *n* = 3.

#### 3.3.3 BT‐1 Induces Changes in Body Shape, Size, and Membrane Permeability

Furthermore, SEM analysis revealed that **BT‐1** treatment induced morphological alterations in the promastigote forms. Untreated parasites showed typical characteristics of normal cells, for example, an elongated shape, a flagellum proportional to body size, and a smooth and intact cell surface (Figure 3A1–A2). However, parasites treated with **BT‐1** for 72 h had a rounded shape and a reduction in cell body size, cell surface roughness, a shorter flagellum, and extravasation of cytoplasmic content (Figure 3A3–A6). Cytometric analysis confirmed a reduction in body size in cells treated with **BT‐1**.

The membrane integrity decreased significantly following treatment with **BT‐1**, as indicated by the increase of PI‐stained cells of 3.48‐ and 11.13‐fold after treatment with IC_50_ and 2 × IC_50_, respectively (Figure 3B3–4), compared with the untreated control (Figure 3B1). Digitonin showed an increase of 3.10‐fold of PI‐stained cells (Figure 3B2). This loss of plasma membrane integrity in **BT‐1**‐treated parasites could result from lipid peroxidation. Additionally, histograms revealed a decrease of 1.45‐fold in cell size after 24‐h treatment with 2 × IC_50_ of **BT‐1** compared with the untreated control (Figure 3C3). Actinomycin D induced a reduction of 2.05‐fold in cell size (Figure 3C1).

#### 3.3.4 BT‐1 Induces Cell Cycle Arrest in *L. amazonensis* Promastigotes

Miltefosine, used as a positive control, notably induced arrest in the sub‐G0 phase of the cell cycle, as evidenced by a 12.33‐fold increase compared with the untreated control (Figure 4A1,A3), indicating DNA fragmentation. Similarly, **BT‐1** treatment resulted in the accumulation of cells in the sub‐G0/G1 and G0/G1 phases, with a 2.22‐ and 1.3‐fold increase, respectively (Figure 4A2,A3).

## 4 Discussion

The urgent need to develop new drugs that are effective, safe, orally bioavailable, and active against parasites that cause leishmaniasis is highlighted by the limited arsenal of pharmacological choices for treating patients, which necessitates prolonged treatment courses and carries significant risks of adverse effects. No vaccines or chemoprophylaxis options are available for humans (Müller et al. 2017; Ponte‐Sucre et al. 2017; Pradhan et al. 2022). This study synthesized and evaluated a series of bithiophene‐based compounds motivated by previous findings demonstrating that thiophene derivatives are potent inhibitors of trypanosomatids (Takahashi et al. 2011; Volpato et al. 2018; Scariot et al. 2019; Poletto et al. 2021).

Before all the in vitro biological tests, *in silico* analyses were performed to evaluate the bioavailability of the compounds and avoid later failure and loose ends, like adverse effects of these molecules (Chagas et al. 2018). Lipinski's RO5 is widely used to predict the molecular drugability of substances (Poletto et al. 2021), either natural or synthetic molecules. For a molecule to be considered an ideal drug, it should adhere to the physicochemical property guidelines outlined by Lipinski's Rule of Five (RO5), which assesses the drug‐likeness of a compound intended for oral administration. According to the RO5, a drug‐like compound should have a molecular weight below 500 g/mol, a log*P* value (indicating hydrophobicity) of less than 5, no more than 5 hydrogen bond donors (HBD), and no more than 10 hydrogen bond acceptors (HBA) (Lipinski et al. 2001; Chagas et al. 2018; Omran and Rauch 2014). Beyond the RO5, polar surface area (PSA) and the number of rotatable bonds (RB) are also important for drug‐likeness, as they correlate with drug permeability and flexibility. An ideal PSA is ≤ 140 Å^2^, and RB should be fewer than 10 (Veber et al. 2002; Matsson et al. 2016). The compounds examined in this study showed no more than one violation, indicating that all of them have good bioavailability.

Thiophenes are considered a versatile class of heterocyclic compounds that possess the characteristics of a privileged structure and have attracted attention in the medicinal field due to their diverse biological activities. Furthermore, Schiff bases are known to exhibit potent biological activities, but despite showing good activity against the parasites, the IC_50_ values of the Schiff bases found in this work showed no difference regardless of the substituent at the imine group. The thiosemicarbazones were expected to demonstrate favorable IC_50_ values; however, the presence of a hydrazinecarbothioamide moiety at the 5‐position of the bithiophene nucleus resulted in no enhancement of activity. However, despite its favorable SI, amphotericin B is known for its significant systemic toxicity, which restricts its therapeutic use and requires careful clinical monitoring (Hamill 2013). This highlights the ongoing need for new compounds with both high efficacy and improved safety profiles.

In summary, among the synthesized compounds, **BT‐1** emerged as the most potent compound; this IC_50_ value represents an improvement over previously studied thiophene compounds evaluated against *L. amazonensis* that showed an IC_50_ of 29.34 and 77.62 μM (Takahashi et al. 2011) warranting further investigation into the mechanism of action of **BT‐1**.

Trypanosomatids, including *Leishmania* spp., possess a distinctive ultrastructure with a single, branched mitochondrion, making this organelle an appealing target for drug development (Menna‐Barreto, Goncalves, et al. 2009). Hypothesizing the mitochondria could be the thiophene target, we investigated morphological, ultrastructural, and biochemical alterations made by **BT‐1**. Electron microscopy (EM) was performed with the same endpoint (72 h) as used for IC_50_ to allow visualization of the final ultrastructural and morphological changes associated with parasite death. EM revealed alterations related to cellular stress, and the accumulation of vesicles in the flagellar pocket likely reflects intense exocytosis of abnormal macromolecules, including deformed proteins and lipids (Dolai and Adak 2014; Halliday et al. 2021). These changes have been found before (De Sarkar et al. 2019; Lazarin‐Bidóia et al. 2022; Soto‐Sánchez 2022) and suggest initiating recovery mechanisms or triggering apoptosis in cases of irreparable damage (da Silva Rodrigues et al. 2019; Menna‐Barreto and de Castro 2014).

In contrast, for biochemical parameters evaluation, a shorter incubation period of 24 h was employed to capture early cellular events leading to death, and 2 × IC_50_ values were used to simulate an exacerbated response. Mitochondrial dysfunction was further confirmed by the detection of oxidative stress and lipid peroxidation, which, in high concentrations of ROS, can degrade macromolecules such as proteins, lipids, and DNA, leading to cellular damage. These damages can include loss of mitochondrial potential and lipid droplets accumulation, composed of neutral fats, triglycerides, and sterols, which are commonly associated with mitochondrial dysfunction and apoptosis (Basmaciyan and Casanova 2019; Das et al. 2021). Along with cell cycle arrest, all these changes in the parasite lead to apoptosis‐like death, the most common cell death in *Leishmania* spp. (Balaña‐Fouce et al. 2014; Sarkar et al. 2024). Although a reduction in cell size was observed, the intense loss of cellular content suggests that necrotic cell death cannot be excluded. Nonetheless, membrane rupture may also reflect late apoptosis, a stage in which cells often become morphologically and functionally indistinguishable from necrotic ones (Basmaciyan and Casanova 2019). These findings raise the possibility that **BT‐1** may, to some extent, induce regulated cell death (RCD) in *L. amazonensis*, a process typically triggered by microenvironmental disruptions such as nonphysiological oxidative stress (Kroemer et al. 2009). In brief, **BT‐1** induced changes consistent with RCD. These findings align with previous studies showing that thiophenic compounds target the mitochondrion in *Leishmania* spp., inducing swelling and depolarization (Takahashi et al. 2011).

Despite *Leishmania* spp. parasites exhibiting two main evolutionary forms—promastigotes and amastigotes (Vannier‐Santos et al. 2002), this pilot study focused solely on promastigotes. Drug screening assays using *Leishmania* promastigotes are widely adopted in early‐stage compound evaluation due to their simplicity, low cost, and scalability. These forms can be cultured in inexpensive liquid media under standard laboratory conditions (25°C–27°C) without the need for host cells, enabling high‐yield, reproducible parasite growth well suited for high‐throughput screening, and some drugs have been identified starting from promastigote‐based screening (Patil et al. 2010; Siqueira‐Neto et al. 2010). In contrast, assays targeting intracellular amastigotes involve infection of macrophage cultures, staining procedures (e.g., Giemsa), and microscopic quantification of parasite burden in hundreds of host cells. This approach is labor‐intensive, time‐consuming, and poorly compatible with automation. Additionally, drug efficacy assessments in amastigotes are more variable due to host cell factors and the inherent challenges of evaluating parasite viability microscopically (Cohen and Azas 2021).

Given these limitations, promastigote‐based assays represent a more practical and cost‐effective option for primary drug screening. Once bithiophenes have been proven effective against *L. amazonensis* parasites, further studies including anti‐amastigote assays, in vivo experiments, and the synthesis of new bithiophene derivatives are underway to further advance this investigation.

In conclusion, this study demonstrates the potential of synthesizing new derivatives based on biologically active chemical nuclei, such as thiophene, to develop novel anti‐leishmanial agents. The effects of **BT‐1** on mitochondrial function highlight its promise as a therapeutic candidate. Future research should focus on optimizing thiophene‐based compounds to improve specificity and efficacy for the treatment of patients with leishmaniasis.

## Conflicts of Interest

The authors declare no conflicts of interest.

## Supporting information


**Data S1:** cbdd70167‐sup‐0001‐sup‐0001‐Supinfo.docx.

## Data Availability

The data that supports the findings of this study are available in the Data S1 of this article.
