# Supplementary material for: Hydroxyalkyne–Bithiophene Derivatives: Synthesis and Antileishmanial Activity
Source: Chem Biol Drug Des. 2025 Aug 22;106(2):e70167. doi: 10.1111/cbdd.70167 (PMC12374030; doi:10.1111/cbdd.70167)
Supplement: Supplementary file 1 — Data S1: cbdd70167‐sup‐0001‐sup‐0001‐Supinfo.docx. [file CBDD-106-e70167-s001.docx]

SUPPLEMENTARY MATERIAL

Hydroxyalkyne-bithiophene derivatives: synthesis and antileishmanial activity

Rayanne Regina Beltrame Machado^a*^, Deysiane Lima Salvador^b*^, Carla Maria Beraldi Gomes^b^, Amanda Beatriz Kawano Bakoshi^a^, Tânia Ueda-Nakamura^a^, Sueli de Oliveira Silva^a^, Celso Vataru Nakamura^a^, Maria Helena Sarragiotto^b#^, Danielle Lazarin-Bidóia^a#^

^a^Laboratório de Inovação Tecnológica no Desenvolvimento de Fármacos e Cosméticos, Departamento de Ciências Básica da Saúde, Universidade Estadual de Maringá (UEM), Maringá, Paraná, Brasil.

^b^Programa de Pós-graduação em Química, Departamento de Química, Universidade Estadual de Maringá (UEM), Maringá, Paraná, Brasil.

*^#^contribute equally

✉[dlbidoia@gmail.com](mailto:dlbidoia@gmail.com)

1. **NMR data of compounds**
   1. **(Hydroxy-alkynyl)-[2,2'-bithiophene]-5-carbaldehydes (1-3)**

5'-(3-Hydroxy-pent-1-ynyl)-[2,2']bithiophenyl-5-carbaldehyde (**1, BT-1**): Yield: 73%. m.p. 80.5 – 81.5 °C. ^1^H NMR (CDCl_3_, 500 MHz,): *δ* = 9.87 (1H, s, CH=O), 7.68 (1H, d, *J* = 4.0 Hz, H-4), 7.25 (1H, d, *J* = 4.0 Hz, H-3’), 7.21 (1H, d, *J* = 4.0 Hz, H-3), 7.15 (1H, d, *J* = 4.0 Hz, H-4’), 4.58 (t, *J* = 6.1 Hz, H-3”), 1.85 (m, H-4”), 1.08 (t, *J* = 7.9 Hz, H-5”). ^13^C NMR (CDCl_3_, 125 MHz,): *δ* = 182.55 (CH=O), 146.02 (C-2), 142.19 (C-5), 137.19 (C-4), 137.09 (C-2), 133.29 (C-4’), 125.80 (C-3), 124.66 (C-3’), 124.14 (C-5’), 96.21 (C-2’’), 77.65 (C-1’’), 64.35 (C-3’’), 30.75 (C-4”), 9.49 (C-5’’).

5'-(4-Hydroxy-but-1-ynyl)-[2,2']bithiophenyl-5-carbaldehyde (**2**): Yield: 80%. m.p. 102.5 – 103.9 °C. ^1^H NMR (CDCl_3_, 500 MHz,): *δ* = 9.88 (1H, s, CH=O), 7.69 (1H, d, *J* = 4.0 Hz, H-4), 7.24 (1H, d, *J* = 4.0 Hz, H-3’), 7.21 (1H, d, *J* = 4.0 Hz, H-3), 7.11 (1H, d, *J* = 4.0 Hz, H-4’), 3.86 (2H, t, *J* = 6.1 Hz, H-4’’), 2.76 (2H, t, *J* = 6.3 Hz, H-3’’); ^13^C NMR (CDCl_3_, 125 MHz,): *δ* = 182.49 (C, CH=O), 146.31 (C-2), 142.12 (C-5), 137.72 (C-4), 136.31 (C-2’), 132.72 (C-4’), 125.77 (C-3), 124.60 (C-5’), 124.51 (C-3’), 93.41 (C-2’’), 75.09 (C-1’’), 60.90 (C-4’’), 24.21 (C-3’’).

5'-(3-Hydroxy-3-methyl-but-1-ynyl)-[2,2']bithiophenyl-5-carbaldehyde (**3**): Yield: 89%. m.p. 105.8 – 106.9 °C. ^1^H NMR (CDCl_3_, 500 MHz,): *δ* = 9.86 (1H, s, CH=O), 7.66 (1H, d, *J* = 4.0 Hz, H-4), 7.23 (1H, d, *J* = 4.0 Hz, H-3’), 7.20 (1H, d, *J* = 4.0 Hz, H-3), 7.12 (1H, d, *J* = 4.0 Hz, H-4’), 1.63 (6H, s, 2CH_3_). ^13^C NMR (CDCl_3_, 125 MHz,): *δ* = 182.55 (C, CH=O), 146.14 (C-2), 142.10 (C-5), 137.25 (C-4), 136.93 (C-2’), 133.12 (C-4’), 125.80 (C-3), 124.61 (C-3’), 124.28 (C-5’), 99.86 (C-2’’), 84.01 (C-3’’), 74.99 (C-1’’), 31.05 (CH_3_).

- 1. **Bithiophene-imines (2a-c) and (3a-c)**

4-(5'-((isopropylimino)methyl)-[2,2'-bithiophen]-5-yl)but-3-yn-1-ol (**2a**): Yield: 38%; m.p. 111.8 – 113.5 °C; ^1^H NMR (CDCl_3_, 500 MHz,): *δ* = 8.32 (1H, s, CH=N), 7.17 (1H, d, *J* = 3.7 Hz, H-4’), 7.12 (1H, d, *J* = 3.7 Hz, H-4’), 7.07 (2H, m, H-3’, H-3), 3.84 (2H, m, H-1”), 3.52 (1H, hept, *J* = 6.7 Hz, CH-isopropyl), 2.74 (2H, t, *J* = 6.1 Hz, H-2”), 1.26 (6H, d, *J* = 6.1 Hz, CH_3_-isopropyl).

4-(5'-((butylimino)methyl)-[2,2'-bithiophen]-5-yl)but-3-yn-1-ol **(2b**): Yield: 55%; m.p. 76.8 – 78.4 °C; ^1^H NMR (CDCl_3_, 500 MHz,): *δ* = 8.29 (1H, s, CH=N), 7.18 (1H, d, *J* = 3.7 Hz, H-4’), 7.12 (1H, d, *J* = 3.7 Hz, H-4), 7.08 (2H, m, H-3’, H-3), 3.84 (2H, t, *J* = 6.7 Hz, H-1”), 3.58 (2H, t, *J* = 7.3 Hz, butyl), 2.74 (2H, t, *J* = 6.7 Hz, H-2”), 1.69 (2H, qt, *J* = 7.3 Hz, butyl), 1.38 (2H, sext., *J* = 7.3 Hz, CH_2_-butyl), 0.96 (3H, t, *J* = 7.3 Hz, butyl); ^13^C NMR (CDCl_3_, 125 MHz,): *δ* = 153.66 (CH=N), 141.63, 139.41, 137.68, 132.58, 130.66, 124.11, 123.89, 123.03, 92.21, 75.46, 61.12, 60.95, 32.94, 24.21, 20.44, 13.89.

4-(5'-((cyclohexylimino)methyl)-[2,2'-bithiophen]-5-yl)but-3-yn-1-ol **(2c):** Yield: 58%; mp 69.3 – 70.5 °C; ^1^H NMR (CDCl_3_, 300 MHz,): *δ* = 8.32 (1H, s, CH=N), 7.15 (1H, d, *J* = 3.7 Hz, H-4’), 7.10 (1H, d, *J* = 3.7 Hz, H-4), 7.06 (2H, m, H-3’, H-3), 3.82 (2H, t, *J* = 6.7 Hz, H-1”), 3.16 (1H, m, cyclohexyl), 2.73 (2H, t, *J* = 6.7 Hz, H-2”), 1.84 – 1.71 (6H, m, cyclohexyl), 1.42 – 1.21 (4H, m, cyclohexyl); ^13^C NMR (CDCl_3_, 75 MHz,): *δ* = 151.73 (C, CH=N), 142.15, 139.45, 137.98, 132.78, 130.80, 124.24, 124.05, 123.15, 92.36, 75.69, 69.83, 61.16, 34.46, 25.77, 25.02, 24.41.

4-(5'-((isopropylimino)methyl)-[2,2'-bithiophen]-5-yl)-2-methylbut-3-yn-2-ol **(3a)**: Yield: 42%; mp 89.2 – 90.0 °C; ^1^H NMR (CDCl_3_, 500 MHz,): *δ* = 8.33 (1H, s, CH=N), 7.16 (1H, d, *J* = 3.7 Hz, H-4’), 7.12 (1H, d, *J* = 3.7 Hz, H-4), 7.08 (2H, m, H-3’, H-3), 3.52 (1H, hept, *J* = 6.1 Hz, CH-isopropyl), 1.63 (6H, s, 2CH_3_), 1.26 (6H, d, *J* = 6.7 Hz, CH_3_-isopropyl); ^13^C NMR (CDCl_3_, 125 MHz,): *δ* = 151.27, 141.88, 139.19, 138.39, 133.00, 130.65, 124.10, 123.95, 122.16, 98.84, 75.40, 65.84, 61.34, 31.30, 24.05.

4-(5'-((butylimino)methyl)-[2,2'-bithiophen]-5-yl)-2-methylbut-3-yn-2-ol **(3b):** Yield: 45%; m.p. 63.4 – 64.3 °C; ^1^H NMR (CDCl_3_, 500 MHz,): *δ* = 8.29 (1H, s, CH=N), 7.17 (1H, d, *J* = 3.7 Hz, H-4’), 7.11 (1H, d, *J* = 3.7 Hz, H-4), 7.08 (2H, m, H-3’, H-3), 3.58 (2H, t, *J* = 7.3 Hz, butyl), 1.68 (2H, qt, *J* = 7.3 Hz, butyl), 1.62 (6H, s, 2CH_3_), 1.38 (2H, sext., *J* = 7.3 Hz, butyl), 0.94 (3H, t, *J* = 7.30 Hz, butyl); ^13^C NMR (CDCl_3_, 125 MHz,): *δ* = 153.66 (C, CH=N), 141.73, 139.31, 138.32, 132.99, 130.69, 124.17, 124.01, 122.23, 98.89, 75.37, 65.82, 61.12, 32.93, 31.30, 20.44, 13.89; HRMS-ESI calcd for C_18_H_21_NOS_2_ [M+H]^+^ 332.1137, found: 332.1140.

4-(5'-((cyclohexylimino)methyl)-[2,2'-bithiophen]-5-yl)-2-methylbut-3-yn-2-ol (**3c):** Yield: 50%; mp 88.9 – 90.1 °C; ^1^H NMR (CDCl_3_, 500 MHz,): *δ* = 8.33 (1H, s, CH=N), 7.15 (1H, d, *J* = 3.7 Hz, H-4’), 7.11 (1H, d, *J* = 3.7 Hz, H-4), 7.06 (2H, m, H-3’, H-3), 3.17 (1H, m, cyclohexyl), 1.85 – 1.73 (6H, m, cyclohexyl), 1.62 (6H, s, 2CH_3_), 1.40 – 1.20 (4H, m, cyclohexyl). ^13^C NMR (CDCl_3_, 125 MHz,): *δ* = 151.5 (C, CH=N), 142.1, 139.1, 138.42, 133.00, 130.59, 124.08, 123.96, 122.13, 98.83, 75.13, 69.62, 65.82, 34.25, 31.29, 25.57, 24.82.

**1.3 Thiosemicarbazones (2d and 3d)**

2-((5’-(4-hydroxybut-1-yn-1-yl)-[2,2’-bithiophen]-5-yl)methylene)hydrazine carbothioamide **(2d)**: Yield: 62%; mp 234.9 – 235.4 °C; ^1^H NMR (DMSO-d_6_, 500 MHz,): *δ* = 11.51 (1H, s, NH), 8.23, 7.61 (2H, s, NH_2_), 8.19 (1H, s, CH=N), 7.41 (1H, d, *J* = 3.7 Hz, H-4), 7.35 (1H, d, *J* = 4.2 Hz, H-4’), 7.30 (1H, d, *J* = 4.2 Hz, H-3’), 7.21 (1H, d, *J* = 3.7 Hz, H-3), 3.57 (2H, t, *J* = 6.7 Hz, H-4’’), 2.61 (2H, t, *J* = 6.7 Hz, H-3’’); ^13^C NMR (DMSO-*d_6_*, 125 MHz,): *δ* = 177.41 (C, H_2_N-C=S), 137.97, 137.29, 136.88 (CH=N), 136.38, 132.86, 131.64, 125.05, 124.67, 122.43, 94.93, 73.80, 59.29, 23.50.

2-((5'-(3-hydroxy-3-methylbut-1-yn-1-yl)-[2,2'-bithiophen]-5-yl)methylene)- hydrazinecarbothioamide **(3d)**: Yield: 52%; mp 198.4 – 199.6 °C; ^1^H NMR (DMSO-d_6_, 500 MHz,): *δ* = 11.53 (1H, s, NH), 8.24, 7.62 (2H, s, NH_2_), 8.19 (1H, s, CH=N), 7.42 (1H, d, *J* = 3.7 Hz, H-4), 7.36 (1H, d, *J* = 4.2 Hz, H-4’), 7.31 (1H, d, *J* = 4.2 Hz, H-3’), 7.24 (1H, d, *J* = 3.7 Hz, H-3), 1.46 (6H, s, 2CH_3_); ^13^C NMR (DMSO-*d_6_*, 125 MHz,): *δ* = 178.00 (H_2_N-C=S), 137.74, 137.53, 137.45, 133.74, 132.23, 125.82, 125.36, 122.14, 102.10, 73.97, 64.32, 31.76; HRMS-ESI calcd for C_15_H_15_N_3_OS_3_ [M+H]^+^ 350.0450, found: 350.0432.

1. **NMR spectra of synthetized compounds**

**Figure S1:** ^1^H NMR spectra (500 MHz, CDCl_3_) of compound **1** **(BT-1**)

**Figure S2:** ^13^C NMR spectra (125 MHz, CDCl_3_) of **BT-1**

**Figure S3:** COSY spectra (500 MHz, CDCl_3_) of **BT-1**

**Figure S4:** HSQC spectra (500 MHz, CDCl_3_) of **BT-1**

**Figure S5:** HMBC spectra (500 MHz, CDCl_3_) of **BT-1**

**Figure S6:**^1^H NMR spectra (500 MHz, CDCl_3_) of compound **2**


**Figure S7:**^13^C NMR spectra (125 MHz, CDCl_3_) of compound **2**

**Figure S8:**^1^H NMR spectra (500 MHz, CDCl_3_) of compound **3**

**Figure S9:**^13^C NMR spectra (125 MHz, CDCl_3_) of compound **3**

**Figure S10:**^1^H NMR spectra (500 MHz, CDCl_3_) of compound **2a**

**Figure S11:**^1^H NMR spectra (500 MHz, CDCl_3_) of compound **2b**

**Figure S12:**^13^C NMR spectra (125 MHz, CDCl_3_) of compound **2b**

**Figure S13:** COSY spectra (500 MHz, CDCl_3_) of compound **2b**

**Figure S14:** HSQC spectra (500 MHz, CDCl_3_) of compound **2b**

**Figure S15:**^1^H NMR spectra (500 MHz, CDCl_3_) of compound **2c**

**Figure S16:**^13^C NMR spectra (125 MHz, CDCl_3_) of compound **2c**

**Figure S17:** COSY spectra (500 MHz, CDCl_3_) of compound **2c**

**Figure S18:** HSQC spectra (500 MHz, CDCl_3_) of compound **2c**

**Figure S19:**^1^H NMR spectra (500 MHz, CDCl_3_) of compound **3a**

**Figure S20:**^13^C NMR spectra (125 MHz, CDCl_3_) of compound **3a**

**Figure S21:** COSY spectra (500 MHz, CDCl_3_) of compound **3a**

**Figure S22:** HSQC spectra (500 MHz, CDCl_3_) of compound **3a**

**Figure S23:**^1^H NMR spectra (500 MHz, CDCl_3_) of compound **3b**

**Figure S24:**^13^C NMR spectra (125 MHz, CDCl_3_) of compound **3b**

**Figure S25:** COSY spectra (500 MHz, CDCl_3_) of compound **3b**

**Figure S26:** HSQC spectra (500 MHz, CDCl_3_) of compound **3b**

**Figure S27:**^1^H NMR spectra (500 MHz, CDCl_3_) of compound **3c**

**Figure S28:**^13^C NMR spectra (125 MHz, CDCl_3_) of compound **3c**

**Figure S29:** COSY spectra (500 MHz, CDCl_3_) of compound **3c**

**Figure S30:** HSQC spectra (500 MHz, CDCl_3_) of compound **3c**

**Figure S31:**^1^H NMR spectra (500 MHz, DMSO-*d_6_*) of compound **2d**

**Figure S32:**^13^C NMR spectra (125 MHz, DMSO-*d_6_*) of compound **2d**

**Figure S33:** COSY spectra (500 MHz, DMSO-*d_6_*) of compound **2d**

**Figure S34:** HSQC spectra (500 MHz, DMSO-*d_6_*) of compound **2d**

**Figure S35:** HMBC spectra (500 MHz, DMSO-*d_6_*) of compound **2d**

**Figure S36:**^1^H NMR spectra (500 MHz, DMSO-*d_6_*) of compound **3d**


**Figure S37:**^13^C NMR spectra (125 MHz, DMSO-*d_6_*) of compound **3d**

**Figure S38:** COSY spectra (500 MHz, DMSO-*d_6_*) of compound **3d**

**Figure S39:** HSQC spectra (500 MHz, DMSO-*d_6_*) of compound **3d**

**Figure S40:** HMBC spectra (500 MHz, DMSO-*d_6_*) of compound **3d**
